# Antipathogenic Potential of a Polyherbal Wound-Care Formulation (Herboheal) against Certain Wound-Infective Gram-Negative Bacteria

**DOI:** 10.1155/2019/1739868

**Published:** 2019-01-27

**Authors:** Pooja Patel, Chinmayi Joshi, Vijay Kothari

**Affiliations:** Institute of Science, Nirma University, Ahmedabad-382481, India

## Abstract

This study investigated antipathogenic efficacy of a polyherbal wound-healing formulation Herboheal against three multidrug-resistant strains of gram-negative bacterial pathogens associated with wound infections. Herboheal was evaluated for its quorum-modulatory potential against three different human-pathogenic bacteria, first *in vitro* through the broth dilution assay and then *in vivo* in the model host *Caenorhabditis elegans*. Herboheal at ≥0.1% v/v was able to inhibit (19–55%) *in vitro* production of quorum sensing-regulated pigments in all these bacteria and seemed to interfere with bacterial quorum sensing by acting as a signal-response inhibitor. This formulation could compromise haemolytic activity of all three bacteria by ∼18–69% and induced their catalase activity by ∼8–21%. Herboheal inhibited *P. aeruginosa* biofilm formation up to 40%, reduced surface hydrophobicity of *P. aeruginosa* cells by ∼9%, and also made them (25%) more susceptible to lysis by human serum. Antibiotic susceptibility of all three bacteria was modulated owing to pretreatment with Herboheal. Exposure of these test pathogens to Herboheal (≥0.025% v/v) effectively reduced their virulence towards the nematode *Caenorhabditis elegans*. Repeated subculturing of *P. aeruginosa* on the Herboheal-supplemented growth medium did not induce resistance to Herboheal in this mischievous pathogen, and this polyherbal extract was also found to exert a post-extract effect on *P. aeruginosa*, wherein virulence of the Herboheal-unexposed daughter cultures, of the Herboheal-exposed parent culture, was also found to be attenuated. Overall, this study indicates Herboheal formulation to be an effective antipathogenic preparation and validates its indicated traditional therapeutic use as a wound-care formulation.

## 1. Introduction

Wound refers to a form of physical injury where the skin is torn, cut, or burned, and it can serve as a site of entry for the pathogenic microbes. Although small, a wound always carries the probability of serving as an entry port for microorganisms, some of whom can complicate the situation further by their biofilm-forming ability. Biofilm formation in chronic wounds presents a difficult challenge in wound management [1]. For effective and rapid healing of a wound, preventing infection in it is essential [2]. Advanced wound care is a big market globally, and many of the leading pharmaceutical firms are actively engaged in this area [3].

Traditional medicines from different geographic locations prescribe a variety of wound-healing formulations, many of which are based on plant extracts and/or oils. A search for plants useful in wound care in the IMPPAT database returns 40 plants (https://cb.imsc.res.in/imppat/Therapeuticsplants/WOUNDS). Since preventing infection is an essential requirement for effective wound healing, we undertook this study to investigate the possible anti-infective potential of a polyherbal formulation indicated for wound healing, namely, “Herboheal,” wherein we studied the effect of this formulation on three different gram-negative bacteria's growth and quorum sensing (QS), besides few other virulence traits. QS is a chemical signal-based process of intercellular communication among bacteria, which regulates expression of many genes including those associated with virulence. Recently, QS is being considered as an important novel target for development of new antipathogenic agents, which in gram-negative bacteria usually employs AHLs (acyl homoserine lactones) as the chemical signal [4]. Pigment production in all three bacteria (*Chromobacterium violaceum*, *Serratia marcescens*, and *Pseudomonas aeruginosa*) used in this study is believed to be regulated by QS [5–8], and association of each of them with wound infections has also been reported. Fatal wound infection caused by *Chromobacterium violaceum* in Vietnam was reported in [9]. Traumatically injured tissues are prone to *P. aeruginosa* wound infection [10]. Involvement of *S. marcescens* in wound and soft tissue infections in patients receiving wound care has also been reported [11]. These pathogens have also been reported to possess a variety of immune evasion mechanisms. Virulence factors like exotoxin A, haemolysin, alkaline protease, elastase, phospholipase C, and biofilm formation enable *P. aeruginosa* to evade the hose immune response. Formation of the alginate layer by this bacterium limits the accessibility of host plasma factors [12]. *S. marcescens* has also been reported to suppress host innate immunity by inducing apoptosis of host immune cells via flagella- and lipopolysaccharide-dependent motility [13]. Ishii et al. showed suppression of host cellular immunity by this pathogen via production of an adhesion-inhibitory factor against immunosurveillance cells [14]. *C. violaceum* exoproteome has also been shown to contain a protein involved in cell adhesion, namely, EF-Tu, which in *P. aeruginosa* allows it to evade the immune system and invade the hose [15]. In face of the multiple immune evasion strategies adopted by these pathogens, investigation on novel formulations capable of curbing their virulence becomes even more important.

## 2. Materials and Methods

### 2.1. Test Formulation

Herboheal formulation (License no. GA/1616) was procured from the SRISTI organization, Ahmedabad. Brief notes on this formulation can be seen at http://www.sristiinnovation.com/human-products-best-for-your-skin.html#herboheal-herbal-wound-healing-ointment. Further details on this formulation including its composition have been provided in the supplementary file (Appendix A). For the purpose of our assays, we took this formulation without one of its bulking agents, i.e., bee wax, as the whole formulation (along with bee wax) was not soluble in the assay media. Bee wax was separately confirmed to have no effect on bacterial growth and pigment production. Before being used for experiments, the test formulation was filtered through a 0.45 *µ*m PVDF membrane filter (Axiva, Haryana).

### 2.2. Test Organisms

*C. violaceum* (MTCC 2656) and *S. marcescens* (MTCC 97) were procured from MTCC (Microbial Type Culture Collection, Chandigarh). *P. aeruginosa* was taken from our own lab's culture collection, whose identity has been confirmed through biochemical tests, and earlier, we had subjected this culture to whole transcriptome sequencing (Bioproject no. PRJNA386078) too. Pseudomonas agar (HiMedia, Mumbai) was used for the maintenance of this culture. *C. violaceum* and *S. marcescens* were grown in the nutrient broth (HiMedia, Mumbai). Incubation temperature for *C. violaceum* and *P. aeruginosa* was 37°C, and for *S. marcescens*, it was 28°C. Incubation time for all three bacteria was kept as 22–24 h. The antibiotic susceptibility profile of the bacterial strains used in this study was generated using the antibiotic discs—Dodeca Universal-I, Dodeca G-III-Plus, and Icosa Universal-2 (HiMedia, Mumbai). *C. violaceum* and *S. marcescens* were found to be resistant to cefadroxil (30 *µ*g), ampicillin (10 *µ*g), cloxacillin (1 *µ*g), and penicillin (10 *µ*g). *S. marcescens* showed resistance against vancomycin (30 *µ*g) too. The strain of *P. aeruginosa* was found to be resistant to amoxicillin (30 *µ*g), cefadroxil (30 *µ*g), ampicillin (10 *µ*g), cloxacillin (1 *µ*g), penicillin (10 *µ*g), chloramphenicol (30 *µ*g), cefixime (5 *µ*g), clindamycin (2 *µ*g), and nitrofurantoin (300 *µ*g).

Two probiotic strains were also used in this study for assessing the prebiotic potential of the test formulation. Of them, *Lactobacillus plantarum* (MTCC 2621) was grown in the Lactobacillus MRS medium (HiMedia, Mumbai), and *Bifidobacterium bifidum* (NCDC 255), procured from the National Collection of Dairy Cultures, Karnal, was grown on MRS agar with 0.05% cysteine.

### 2.3. Broth Dilution Assay

Assessment of QS-regulated pigment production by test pathogens in presence or absence of the test formulation was done using the broth dilution assay [16]. Organisms were challenged with different concentrations (0.05–1% v/v) of Herboheal formulation (HF). Nutrient broth or pseudomonas broth (peptic digest of animal tissue 20 g/L, potassium sulphate 10 g/L, and magnesium chloride 1.4 g/L; pH 7.0 ± 0.2) was used as a growth medium. Inoculum standardized to 0.5 McFarland turbidity standard was added at 10% v/v, to the media supplemented with required concentration of HF, followed by incubation at appropriate temperature for each organism. Abiotic control (containing the extract and growth medium, but no inoculum) was also included in the experiment. Catechin (50 *μ*g/mL; Sigma-Aldrich, USA) was used as a positive control.

### 2.4. Measurement of Bacterial Growth and Pigment Production

At the end of the incubation, bacterial growth was quantified at 764 nm [17]. This was followed by pigment extraction and quantification, as per the method described below for each of the pigment. Purity of each of the extracted pigment was confirmed by running a UV-Vis scan (Agilent Cary 60 UV-visible spectrophotometer). Appearance of a single major peak (at *λ*_max_ reported in the literature) was taken as indication of purity.

#### 2.4.1. Violacein Extraction

One millilitre of the *C. violaceum* culture broth was centrifuged (Eppendorf 5417R) at 15,300 g for 10 min at room temperature, and the resulting supernatant was discarded [18]. The remaining cell pellet was resuspended into 1 mL of DMSO and vortexed, followed by centrifugation at 15,300 g for 10 min. The purple-coloured violacein was extracted from the supernatant; OD was measured at 585 nm. Violacein unit was calculated as OD_585_/OD_764_. This parameter was calculated to nullify the effect of any change in cell density on pigment production.

#### 2.4.2. Prodigiosin Extraction

One millilitre of the *S. marcescens* culture broth was centrifuged at 10,600 g for 10 min [19]. Centrifugation was carried out at 4°C, as prodigiosin is a temperature-sensitive compound. The resulting supernatant was discarded. The remaining cell pellet was resuspended into 1 mL of acidified methanol (4 mL of HCl into 96 mL of methanol; Merck), followed by incubation in dark at room temperature for 30 min. This was followed by centrifugation at 10,600 g for 10 min at 4°C. Prodigiosin was obtained from the resulting supernatant; OD was taken at 535 nm. Prodigiosin unit was calculated as OD_535_/OD_764_.

#### 2.4.3. Pyoverdine and Pyocyanin Extraction

One millilitre of the culture broth was mixed with chloroform (Merck, Mumbai) in 2 : 1 proportion followed by centrifugation at 12,000 rpm (15,300 g; REMI CPR-24 Plus) for 10 min [20, 21]. This resulted in formation of two immiscible layers. OD of the upper water-soluble phase containing yellow-green fluorescent pigment pyoverdine was measured at 405 nm. Pyoverdine unit was calculated as OD_405_/OD_764_.

The lower chloroform layer containing pyocyanin was mixed with 0.1 N HCl (Merck; at the rate of 20% v/v), resulting in a colour change from blue to pink. Absorbance of this pyocyanin in the acidic form was measured at 520 nm. Pyocyanin unit was calculated as OD_520_/OD_764._

### 2.5. AHL Augmentation Assay

This assay was done to investigate whether the test formulation exerts its QS-inhibitory effect by inhibiting production of the signal AHL, or by interfering with the signal-response mechanism. Extraction of the *N*-acyl-homoserine lactone (AHL) was performed as described in [22]. OD of the overnight grown bacterial culture was standardized to 1.00 at 764 nm. It was centrifuged at 5000 g for 5 min. Cell-free supernatant was filter-sterilized using a 0.45 *µ*m filter (Axiva, Haryana) and was mixed with equal volume of acidified ethyl acetate (0.1% formic acid (Merck) in ethyl acetate (Merck)). The ethyl acetate layer was collected and evaporated at 55˚C, followed by reconstitution of the dried crystals in 100 *µ*L phosphate buffer saline (pH 6.8). Identity of thus-extracted AHL was confirmed by thin-layer chromatography (TLC) [23]. The *R*_f_ value of purified AHL from the *C. violaceum* culture while performing TLC (methanol (60) : water (40); TLC Silica gel 60 F_254_ plates; Merck) was found to be 0.70, near to that (0.68) reported for *N*-hexonylhomoserine lactone (C6-HSL) [24]. The *R*_f_ value of purified AHL from *S. marcescens*, using the same TLC conditions as mentioned above, was found to be 0.83, near to that (0.80) reported for C12-HSL [25]. TLC of AHL extracted from *P. aeruginosa* resulted in three spots corresponding to *R*_f_ values of 0.43, 0.68, and 0.92 near to those (0.41, 0.68, and 0.84) reported for C8-HSL, C6-HSL, and C4-HSL respectively [24].

The bacterial culture growing in presence of test formulation was supplemented with 2% v/v AHL after 6 hours of incubation, and at the end of a total 24-hour incubation, pigments were extracted from AHL-supplemented experimental tubes, as well as AHL-nonsupplemented control tubes. If the QS-regulated pigment production is found inhibited in both these tubes in comparison to bacteria growing in absence of the extract as well as AHL, then the effect of the test extract was interpreted as a signal-response inhibitor because if the test formulation would have acted as a signal-supply inhibitor, then exogenous supply of AHL should restore pigment formation by the bacteria.

### 2.6. Hemolysis Assay

OD_764_ of the overnight grown (in presence or absence of HF) culture was standardized to 1.00. Cell-free supernatant was prepared by centrifugation at 15,300 g for 10 min [26]. 10 *μ*L of human blood (collected in a heparinized vial) was incubated with this cell-free supernatant for 2 h at 37°C, followed by centrifugation at 800 g for 15 min. OD of the supernatant was read at 540 nm, to quantify the amount of hemoglobin released. 1% Triton X-100 (CDH, New Delhi) was used as a positive control. Phosphate buffer saline was used as a negative control.

### 2.7. Assay of Bacterial Susceptibility to Lysis in Presence of Human Serum

Serum was separated by centrifuging blood at 1,500 rpm (800 g) for 10 min [27]. The bacterial culture grown in media with and without HF was centrifuged, and the cell pellet was reconstituted in PBS so that the resulting suspension attains OD_764_ = 1. 200 *µ*l of this bacterial suspension from control or experimental tubes was mixed with 740 *µ*l of PBS and 60 *µ*l of serum. After incubation for 24 h at 37°C, absorbance was read at 764 nm. The culture not exposed to HF incubated with human serum served as a control, against which OD (post-serum exposure) of the HF-treated cells was compared. Tubes containing bacterial cells (exposed neither to HF nor to serum) suspended in PBS were also included in the experimental setup, to nullify any interference from autolysis.

### 2.8. Catalase Assay

OD_764_ of the culture was adjusted to 1.00. 400 *µ*L of phosphate buffer was added into a 2 mL vial followed by 400 *µ*L of H_2_O_2._ To this, 200 *µ*L of the bacterial culture was added, and the mixture was incubated for 10 min. Then, 10 *µ*M of sodium azide (20 *µ*L) was added to stop the reaction [28], followed by centrifugation at 12,000 rpm for 10 min. OD of the supernatant was measured at 240 nm to quantify the remaining H_2_O_2_ [29], with phosphate buffer as the blank.

### 2.9. Assay for Biofilm Formation, Eradication, and Viability

In this assay, both control and experimental groups contained nine test tubes. In each group, three subgroups were made. The first subgroup of three test tubes in the experimental group contained Pseudomonas broth supplemented with HF, whereas the remaining six tubes contained Pseudomonas broth with no HF on the first day of the experiment. All these tubes were inoculated with inoculum (10% v/v) standardized to 0.5 McFarland turbidity standard (making total volume in a 1 mL tube), followed by incubation at 37°C for 24 h under static condition, which resulted in formation of biofilm as a ring on walls of the glass tubes. This biofilm was quantified by the crystal violet assay [30], preceded by quantification of the bacterial cell density and pigment. Contents from the remaining six test tubes from rest of the two subgroups were discarded following cell density and pigment estimation, and then the biofilms remaining on the inner surface of these tubes were washed with phosphate buffer saline (PBS; pH 7) to remove loosely attached cells. Now, 2 mL of minimal media (sucrose 15 g/L, K_2_HPO_4_ 5.0 g/L, NH_4_Cl 2 g/L, NaCl 1 g/L, MgSO_4_ 0.1 g/L, and yeast extract 0.1 g/L; pH 7.4 ± 0.2) containing HF was added into each of these tubes so as to cover the biofilm completely, and tubes were incubated for 24 h at 37°C. At the end of incubation, one subgroup of 3 tubes was subjected to the crystal violet assay to know whether any eradication of the preformed biofilm has occurred under the influence of HF, and the last subgroup of 3 tubes was subjected to viability assessment through the MTT assay. For the crystal violet assay, the biofilm-containing tubes (after discarding the inside liquid) were washed with PBS in order to remove all nonadherent (planktonic) bacteria and air-dried for 15 min. Then, each of the washed tubes was stained with 1.5 mL of 0.4% aqueous crystal violet solution for 30 min. Afterwards, each tube was washed twice with 2 mL of sterile distilled water and immediately destained with 1500 *μ*L of 95% ethanol. After 45 min of destaining, 1 mL of destaining solution was transferred into separate tubes and read at 580 nm. For the MTT assay [31], the biofilm-containing tubes (after discarding the inside liquid) were washed with PBS in order to remove all nonadherent (planktonic) bacteria and air-dried for 15 min. Then, 900 *µ*L of minimal media was added into each tube, followed by addition of 100 *μ*L of 0.3% MTT (3-(4,5-dimethylthiazol-2-yl)-2,5-diphenyltetrazolium bromide; HiMedia). After 2 h incubation at 37°C, resulting purple formazan derivatives were dissolved in DMSO and measured at 560 nm.

### 2.10. Cell Surface Hydrophobicity (CSH) Assay

Bacterial surface hydrophobicity was measured using the bacterial adhesion to hydrocarbon (BATH) assay as described by Hui and Dykes [32]. The *P. aeruginosa* culture was collected at the stationary phase and pelleted by centrifugation (NF800R; NUVE, Belgium) at 7,000 rpm for 10 min. This pellet was washed twice with phosphate buffer saline (PBS; pH 7.4) and then resuspended in PBS with HF (0.5% v/v) to OD_764_ = 1.00. The same procedure was repeated with the *P. aeruginosa* culture without HF, as a control. Each bacterial suspension was then incubated for 1 h at room temperature. 2 mL sample of each suspension was collected, and absorbance (*A*) at 764 nm was measured, using PBS as the blank. 1 mL of xylene (HiMedia, Mumbai) was added to the 2 mL cell suspension, and this mixture was vortexed for 2 min. The phases were then allowed to separate for 1 h. The absorbance of the aqueous phase (*A*_0_) was again determined. The results were expressed as follows:

(1) $$\begin{array}{c} \%\text{ attachment to xylene}=\left(1-\;\left(\frac{A}{A_{0}}\right)\right)\times 100. \end{array}$$

### 2.11. Determination of the Effect of HF on Antibiotic Susceptibility of the Test Organisms

After *in vitro* assessment of the QS-inhibitory property of the test formulation, the effect of this HF on antibiotic susceptibility of the test pathogen was investigated. The bacterial cells pretreated with HF were subsequently challenged with sub-MIC concentrations of different antibiotics. All the antibiotics were procured from HiMedia, Mumbai.

### 2.12. In Vivo Assay

*In vivo* efficacy of the HF was evaluated using the nematode worm *Caenorhabditis elegans* as the model host, using the method described by Eng and Nathan [33] with some modification. This worm was maintained on the nematode growing medium (NGM; 3 g/L NaCl, 2.5 g/L peptone, 1 M CaCl_2_, 1 M MgSO_4_, 5 mg/mL cholesterol, 1 M phosphate buffer with pH 6, and 17 g/L agar-agar) with *E. coli* OP50 (procured from LabTIE B.V., JR Rosmalen, Netherlands) as the feed. Worm population to be used for the *in vivo* assay was kept on NGM plates not seeded with *E. coli* OP50 for three days, before being challenged with the test pathogen.

Test bacterium was incubated with the HF for 22–24 h. Following incubation, OD_764_ of the culture suspension was equalized to that of the control (not exposed to HF). 100 *μ*L of this bacterial suspension was mixed with 900 *μ*L of the M9 buffer containing 10 worms (L3-L4 stage). This experiment was performed in 24-well (sterile, nontreated) polystyrene plates (TPG24; HiMedia), and incubation was carried out at 22°C. The number of live vs. lead worms was counted everyday till five days by placing the plate (with lid) under light microscope (4x). Standard antibiotics- and catechin-treated bacterial suspension were used as a positive control. Straight worms were considered to be dead. On the last day of the experiment, when plates could be opened, their death was also confirmed by touching them with a straight wire, wherein no movement was taken as confirmation of death.

### 2.13. Statistical Analysis

All the experiments were performed in triplicate, and measurements were reported as mean ± standard deviation (SD) of 3 independent experiments. Statistical significance of the data was evaluated by applying the *t*-test using Microsoft Excel®. *P* values ≤ 0.05 were considered to be statistically significant.

## 3. Results

### 3.1. *C. violaceum*

**Figure 1:**
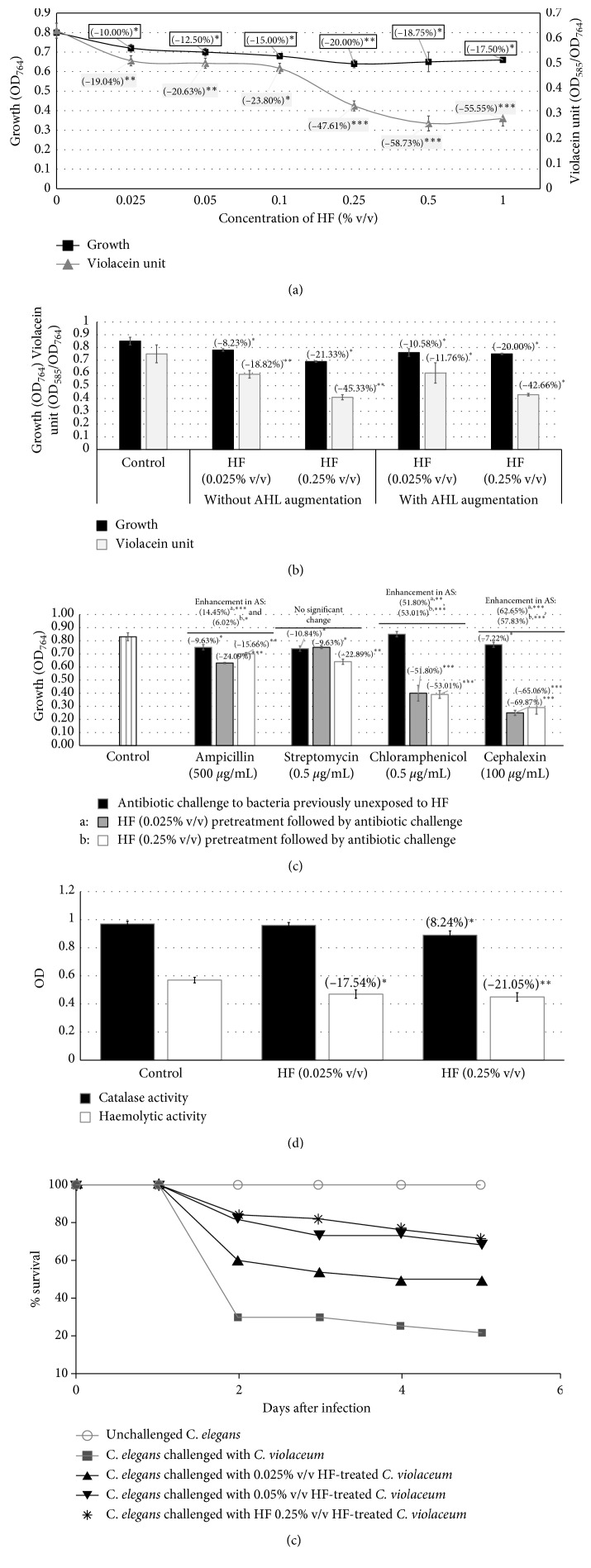
Effect of HF on *C. violaceum*. (a) Effect of HF on growth and QS-regulated violacein production in *C. violaceum*: bacterial growth was measured as OD_764_, OD of violacein was measured at 585 nm, and violacein unit was calculated as the ratio OD_585_/OD_764_ (an indication of violacein production per unit of growth). Catechin (50 *µ*g/mL) did not exert any effect on growth of *C. violaceum* and inhibited violacein production by 47.69%^*∗∗∗*^ ± 0.03. (b) HF acts as a *signal-response inhibitor* against *C. violaceum.* (c) HF pretreatment enhances susceptibility of *C. violaceum* to different antibiotics. (d) HF enhances catalase activity and inhibits haemolytic activity of *C. violaceum*: the catalase assay was done by monitoring disappearance of H_2_O_2_ at 240 nm. Chloramphenicol (0.5 *μ*g/mL) enhanced catalase activity of this bacterium by 11.23%^*∗*^± 0.01. Hemoglobin concentration was measured at OD_540_. (e) HF treatment attenuates virulence of *C. violaceum* towards *C. elegans:* catechin (50 *μ*g/mL) and ampicillin (500 *μ*g/mL) employed as positive controls conferred 100% protection, and HF at 0.025% v/v, 0.05% v/v, and 0.25% v/v conferred 28%^*∗∗*^, 46%^*∗∗∗*^, and 50%^*∗∗∗*^ survival benefit, respectively. Survival benefit refers to the difference between the number of worms surviving in experimental and control wells. HF at tested concentrations showed no toxicity towards the worm. ^*∗*^ *p* < 0.05; ^*∗∗*^ *p* < 0.01; ^*∗∗∗*^ *p* < 0.001; AS: antibiotic susceptibility; QS: quorum sensing; HF: Herboheal formulation.

*C. violaceum* was challenged with 0.025–1% v/v concentration of the HF. All the test concentrations were able to exert the inhibitory effect on *C. violaceum* growth as well as production of QS-regulated pigment violacein, with latter getting affected more than the former (Figure 1(a)). Interestingly, a 40-fold increase in HF concentration (from 0.025 to 1% v/v) could cause only 2.91-fold more inhibition of violacein and only a 1.75-fold higher inhibition of growth. Exogenous addition of the QS signal (AHL) to the quorum-inhibited culture of *C. violaceum* was not found to reverse the inhibitory effect of HF on violacein production (Figure 1(b)), which suggests HF to act as a *signal-response inhibitor* of the QS machinery of this bacterium.

Pretreatment of *C. violaceum* before being challenged with sub-MIC concentrations of four different antibiotics enhanced its susceptibility to all of them, particularly to chloramphenicol and cephalexin (Figure 1(c)). Pretreatment of bacteria with higher (0.25% v/v) HF concentration was not found to modulate the antibiotic susceptibility to any notably greater extent (except in case of streptomycin), than pretreatment with lower (0.025% v/v) concentration. At both these test concentrations, HF was found to compromise the haemolytic potential of *C. violaceum* to a statistically identical extent; however, catalase activity was found to be modulated only at 0.25% v/v (Figure 1(d)). The *in vivo* assay reveled the ability of HF to confer protection on *C. elegans* in face of the *C. violaceum* challenge, wherein the magnitude of survival benefit was observed to increase with the dose of HF (Figure 1(e)). Worm population in the well corresponding to 0.25% v/v HF treatment was also able to generate progeny.

### 3.2. *S. marcescens*

**Figure 2:**
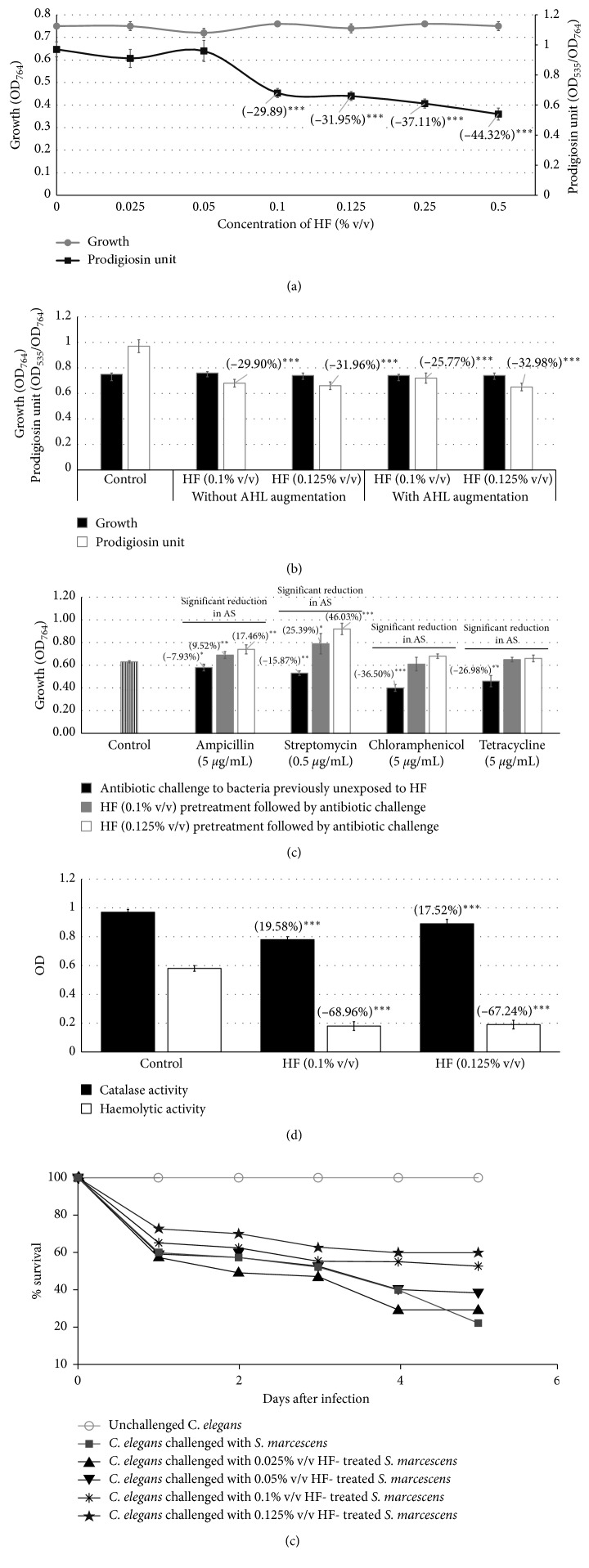
Effect of HF on *S. marcescens.* (a) Effect of HF on growth and QS-regulated prodigiosin production in *S. marcescens*: bacterial growth was measured as OD_764_, OD of prodigiosin was measured at 535 nm, and prodigiosin unit was calculated as the ratio OD_535_/OD_764_ (an indication of prodigiosin production per unit of growth). Catechin (50 *µ*g/mL) inhibited prodigiosin production by 10%^*∗*^ ± 0.05 without affecting bacterial growth. (b) HF acts as a *signal-supply inhibitor* against *S. marcescens*. (c) HF pretreatment reduces susceptibility of *S. marcescens* to different antibiotics. (d) HF enhances catalase activity and inhibits haemolytic activity of *S marcescens*: the catalase assay was done by monitoring disappearance of H_2_O_2_ at 240 nm. Chloramphenicol (5 *μ*g/mL) enhanced catalase activity of this bacterium by 13.40%^*∗*^ ± 0.02. Hemoglobin concentration was measured at OD_540_. (e) HF treatment reduces virulence of *S. marcescens* towards *C. elegans*: catechin (50 *μ*g/mL) and ofloxacin (0.1 *μ*g/mL) employed as positive controls conferred 100% and 80% protection, respectively, on worm population. Pretreatment of bacteria with HF at 0.025%, 0.05%, 0.1%, and 0.125% conferred 7.5%^*∗∗∗*^, 15%^*∗∗∗*^, 30%^*∗∗∗*^, and 37.5%^*∗∗∗*^ survival benefit, respectively. Survival benefit refers to the difference between the number of worms surviving in experimental and control wells. HF at tested concentrations showed no toxicity towards *C. elegans*. ^*∗*^ *p* < 0.05; ^*∗∗*^ *p* < 0.01; ^*∗∗∗*^ *p* < 0.001; AS: antibiotic susceptibility; QS: quorum sensing; HF: Herboheal formulation.

HF till 0.05% v/v exerted no effect on pigment production in *S. marcescens* and an inhibitory effect at higher concentrations, with no effect on growth at any of the concentrations tested (Figure 2(a)). HF's effect on prodigiosin production by *S. marcescens* can be said to follow the threshold model of the dose-response relationship [34]; however, a 5-fold increase in concentration (from 0.1 to 0.5% v/v) resulted in only 1.48-fold higher inhibition of pigment production. This inhibition was not found to reverse upon augmentation of the quorum-inhibited *S. marcescens* culture with AHL, indicating HF to act as a *signal-response inhibitor* against this bacterium too (Figure 2(b)).

Pretreatment of *S. marcescens* with HF reduced its susceptibility to all four antibiotics tested. In fact, HF pretreatment made *S. marcescens* facing the antibiotic challenge grow either at par or even better than the control (not exposed to any antibiotic) *S. marcescens* culture (Figure 2(c)). Catalase activity of *S. marcescens* experienced a promotion, whereas haemolytic activity was heavily compromised under the influence of HF (Figure 2(d)). HF-treated *S. marcescens* could kill lesser number of *C. elegans*, as compared to HF-unexposed control *S. marcescens* (Figure 2(e)).

### 3.3. *P. aeruginosa*

**Figure 3:**
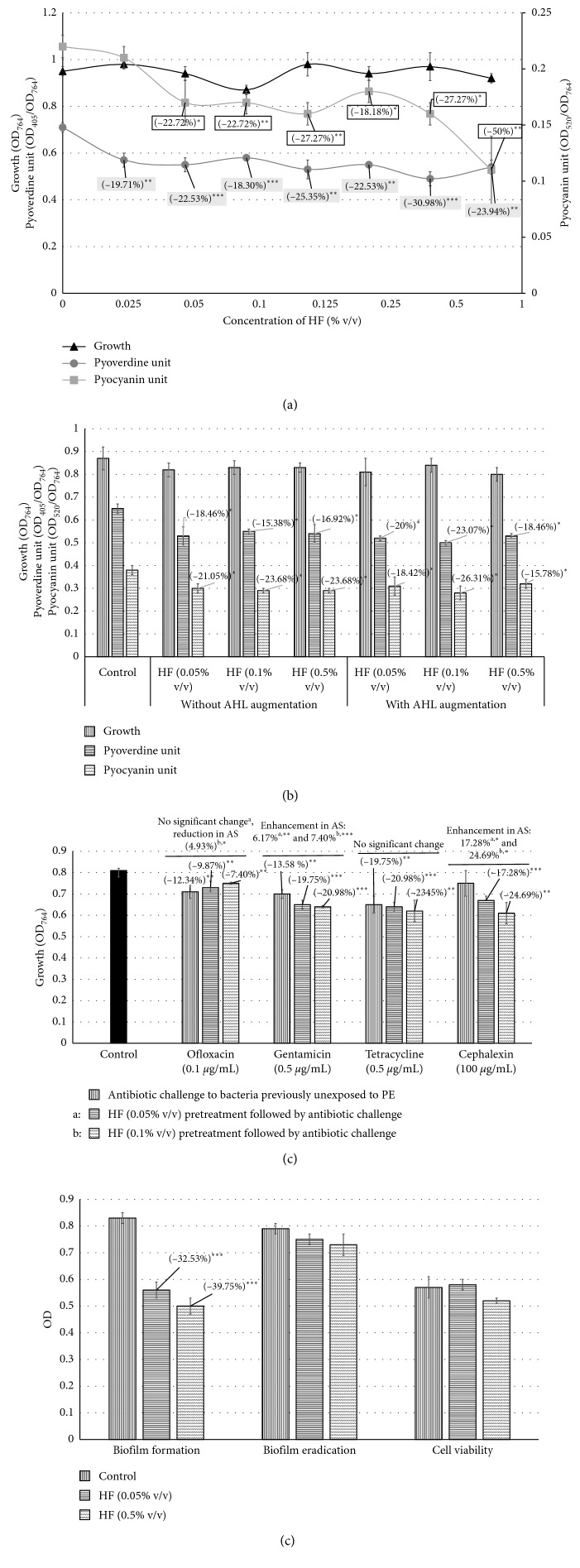
Effect of HF on *P. aeruginosa* growth, pigment production, antibiotic susceptibility, and biofilm. (a) Effect of HF on growth and QS-regulated pigment production in *P. aeruginosa*: bacterial growth was measured as OD_764_, OD of pyoverdine was measured at 405 nm, and OD of pyocyanin was measured at 520 nm. Pyoverdine unit was calculated as the ratio OD_405_/OD_764_ (an indication of pyoverdine production per unit of growth). Pyocyanin unit was calculated as the ratio OD_520_/OD_764_ (an indication of pyocyanin production per unit of growth). Catechin (50 *µ*g/mL) inhibited pyoverdine 17.13%^*∗∗*^ ± 0.06 and pyocyanin 23.65%^*∗*^ ± 0.04 production without affecting the bacterial growth. (b) HF acts as a *signal-response inhibitor* against *P. aeruginosa.* (c) HF treatment made *P. aeruginosa* more susceptible to gentamicin and cephalexin. (d) HF reduced *P. aeruginosa* biofilm formation but did not eradicate preformed biofilm nor had any effect on biofilm viability: the crystal violet assay was performed to measure biofilm formation and biofilm eradication, followed by the measurement of OD at 580 nm. Cell viability in biofilm was estimated through the MTT assay, wherein OD was measured at 560 nm. ^*∗*^ *p* < 0.05; ^*∗∗*^ *p* < 0.01; ^*∗∗∗*^ *p* < 0.001; AS: antibiotic susceptibility; QS: quorum sensing; HF: Herboheal formulation.

HF at any of the concentrations employed did not exert any growth inhibitory effect on *P. aeruginosa*, while negatively affecting production of both the QS-regulated pigments negatively. The magnitude of the pigment inhibitory effect at majority of the HF concentrations was almost similar (Figure 3(a)), and this inhibition was not reversed following AHL augmentation, suggesting HF to act as a *signal-response inhibitor* (Figure 3(b)). Susceptibility of *P. aeruginosa* to gentamicin and cephalexin was raised owing to HF pretreatment, whereas that against ofloxacin and tetracycline remained unaffected (Figure 3(c)). Under the influence of HF, biofilm-forming ability of *P. aeruginosa* was suppressed up to ∼40%; however, this formulation had no effect on preformed biofilm (Figure 3(d)). HF (0.5% v/v) could reduce CSH of *P. aeruginosa* by 9.32% (*p*=0.01), wherein percent hydrophobicity of bacterial cells exposed to HF and control was found to be 21.22% and 30.55%, respectively.

**Figure 4:**
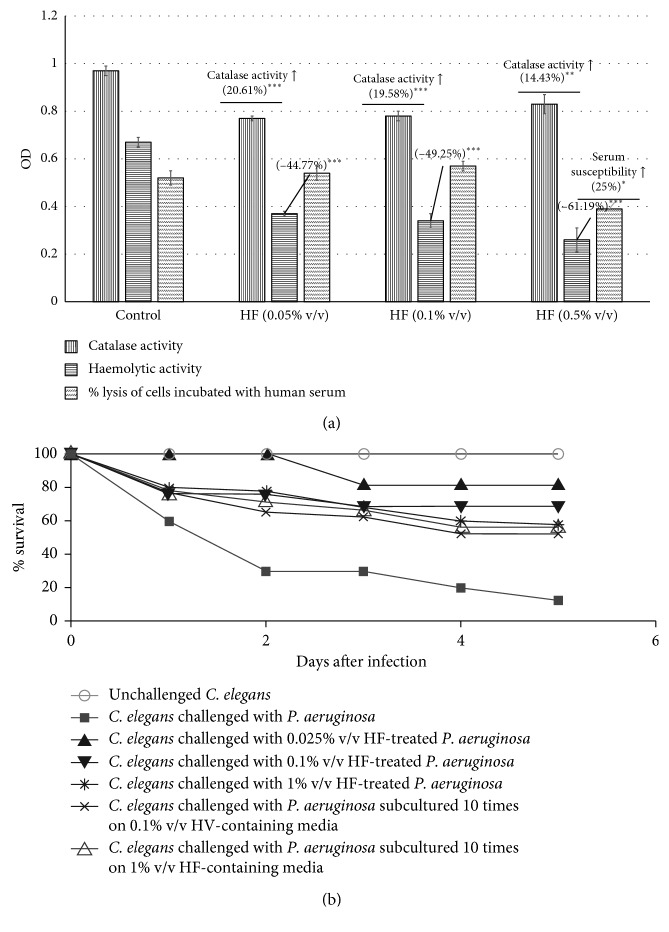
(a) HF enhances catalase activity, inhibits haemolytic potential, and increases susceptibility of *P. aeruginosa* to lysis by human serum: the catalase assay was done by monitoring disappearance of H_2_O_2_ at 240 nm. Tetracycline (0.5 *μ*g/mL) inhibited catalase activity of this bacterium by 21.51%^*∗*^ ± 0.02. Hemoglobin concentration was measured at OD_540_. “Control” in the serum-dependent lysis assay was HF-unexposed cells of *P. aeruginosa* incubated with human serum. (b) HF treatment reduces the virulence of *P. aeruginosa* towards *C. elegans*: catechin (50 *μ*g/mL) and gentamicin (0.1 *μ*g/mL) employed as positive controls conferred 100% and 80% protection, respectively. Pretreatment of bacteria with HF at 0.025% v/v, 0.1% v/v, and 1% v/v conferred 70%^*∗∗∗*^, 55%^*∗∗∗*^, and 45%^*∗∗∗*^ survival benefit, respectively. Survival benefit refers to the difference between the number of worms surviving in experimental and control wells. HF at tested concentrations showed no toxicity towards *C. elegans.* ^*∗*^ *p* < 0.05; ^*∗∗*^ *p* < 0.01; ^*∗∗∗*^ *p* < 0.001; HF: Herboheal formulation.

Exposure to HF induced catalase activity and restricted the haemolytic activity of *P. aeruginosa.* HF treatment could also make *P. aeruginosa* more prone to lysis in presence of human serum (Figure 4(a)). HF-exposed bacteria were able to kill lesser *C. elegans* worms as compared to the HF-unexposed *P. aeruginosa* (Figure 4(b)). The onset of death in worm population was also delayed in the wells corresponding to 0.025% v/v HF concentration, whereas generation of progeny worms was observed in the wells corresponding to 0.1% v/v HF. Paradoxically, pretreatment of bacteria with lower HF concentration attenuated their virulence towards *C. elegans* more than the higher concentration.

**Figure 5:**
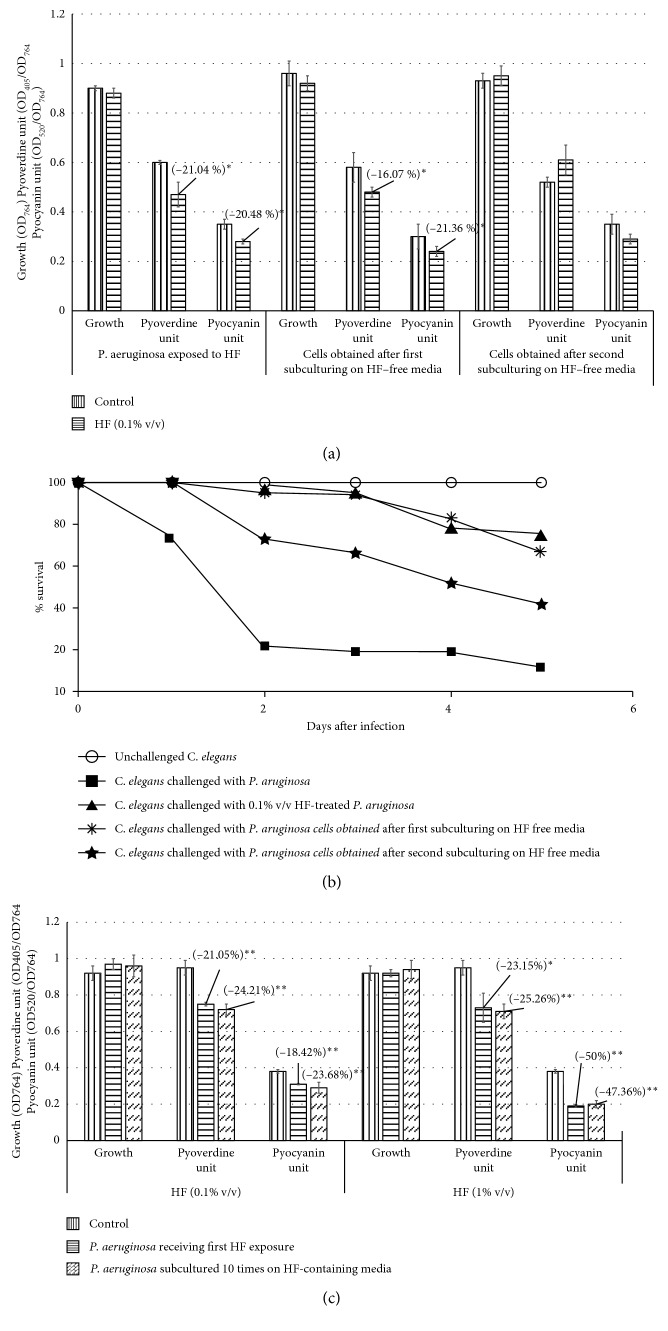
Demonstration of the PEE of HF on *P. aeruginosa in vitro* (a) and *in vivo* (b). (c) Effect of HF on *P. aeruginosa* growth, and pigment production remained unaltered even after repeated exposure to HF: *in vivo* data corresponding to (c) are part of Figure 4(b). ^*∗*^ *p* < 0.05; ^*∗∗*^ *p* < 0.01; HF: Herboheal formulation; PEE: post-extract effect.

After confirming efficacy of HF against *P. aeruginosa in vitro* as well as *in vivo*, we subcultured the HF-treated *P. aeruginosa* in HF-free media to know whether there is any post-exposure residual effect of this formulation on next generations of bacteria. The *P. aeruginosa* culture obtained after one such subculturing on HF-free media was still altered with respect to pyocyanin and pyoverdine production, and the magnitude of this alteration was almost identical to that of *P. aeruginosa* receiving first exposure to HF. This pigment-modulatory effect was not observed in the *P. aeruginosa* culture obtained after second subculturing on HF-free media (Figure 5(a)). Such a long-lasting effect of any antimicrobial observed following a transient exposure of the parent culture to some extracts can be referred as the post-extract effect (PEE) [35–37]. Although this PEE seemed to disappear following second subculturing *in vitro*, the *in vivo* assay revealed that it did not disappear completely. *P. aeruginosa* obtained after second subculturing on HF-free media was still able to kill lesser worms that the HF-unexposed bacterial culture (Figure 5(b)).

In general, repeated exposure of a particular bacterial population to any given antimicrobial agent is expected to exert a strong selection pressure on the bacteria to develop resistant phenotypes. Whether this happens with *P. aeruginosa*, in face of continuous exposure to HF, was also investigated by us (Figure 5(c)). *P. aeruginosa* subcultured ten times on HF-containing media was still not able to kill as many worms as the HF-unexposed bacteria (Figure 4(b)), leading us to conclude that even after repeated exposure to this polyherbal formulation, *P. aeruginosa* could not become resistant to it.

## 4. Discussion

Herboheal formulation was able to exert a quorum-inhibitory effect on all three gram-negative bacteria employed in this study, and it was found to exert its inhibitory effect by interfering with the signal-response part of the QS phenomenon, in all three cases. Since QS machinery of different gram-negative bacteria is believed to have a significant overlap [38], it can be expected from a quorum inhibitor effective against one gram-negative bacteria to be also effective against many other gram-negative bacteria. HF was indicated to act as a signal-response inhibitor against all three gram-negative bacteria used in this study. Since the use of AHLs as QS signals and role of LuxR analogues in mounting AHL-mediated response are common among gram-negative bacteria [38], HF can be expected to act as a broad-spectrum QS inhibitor against multiple gram-negative bacterial pathogens. HF could inhibit *in vitro* production of QS-regulated pigments in *C. violaceum* and *P. aeruginosa* at a concentration as low as 0.025% v/v and at 0.1% v/v in *S. marcescens.* It did not inhibit growth of *S. marcescens* and *P. aeruginosa* at any of the concentrations tested. In fact, it is expected from an ideal antivirulence agent not to have any notable inhibitory effect on growth of susceptible bacteria, which may make them exert lesser selection pressure on the target bacterial populations. Bactericidal antimicrobial agents are believed to apply a heavy selection pressure on the target bacteria by keeping them in a “mutate or die” situation. The newer concept of antipathogenic compounds focuses largely on disarming the pathogens without exerting a sharp killing effect on them. In contrast to the conventional microbicidal antibiotics, antivirulence agents curb the pathogenicity/virulence without necessarily displaying any notable growth-inhibitory effect. Such “pathoblockers” are likely to emerge as a new category of “tailored spectrum” superior therapeutics [39–41]. Based on this logic and early enthusiasm surrounding QS inhibitors, latter were viewed as “evolution-proof drugs” [42]. Although this enthusiasm may not be supported fully by experimental evidence, as reports on resistance to QS inhibitors [43–45] have also appeared in the literature, development of resistance against QS modulators can still be expected to emerge at a relatively slower pace. QS-targeting chemotherapeutic agents are believed to be less likely to generate resistance among pathogenic microbial populations as they target the adaptation and not the survival mechanisms of the pathogen [46, 47]. Treatment of bacteria used in this study by HF at 0.025–0.1% v/v was able to attenuate bacterial virulence enough so that at least 50% of the worms could not be killed in face of the bacterial challenge, as against 77.5–85% killing of worms by HF-unexposed bacteria. Besides challenging the nematode worm *C. elegans* with HF-treated bacteria, we also tested HF as a post-infection therapeutic by offering it to the already-infected *C. elegans*, wherein we allowed *P. aeruginosa* to establish infection for 6 hours or 24 hours before HF was applied (Table S1). In this modified *in vivo* assay too, HF was found to exert its antipathogenic effect notably.

While investigating antibiotic susceptibility of HF-exposed bacteria, in 10 out of 11 such experiments (not considering experiments at two different concentrations of HF as separate), we found antibiotic susceptibility was modulated following HF treatment. *C. violaceum* and *P. aeruginosa* were found to become more susceptible to cephalexin owing to HF pretreatment. Formulations like HF which can extend utility of the cephalexin type of broad-spectrum antibiotics assume importance in face of the fact that cephalexin is reserved as a third-line treatment for certain conditions, e.g., urinary tract infection in pregnant women (https://bpac.org.nz/BPJ/2011/december/cephalosporins.aspx). Since in these experiments only a fraction of the bacterial cells from HF-containing tubes was transferred to the tubes containing antibiotic, the observed modulation of susceptibility can better be explained in the context of PEE. This is based on the understanding that when OD was taken as an end-point measurement in antibiotic susceptibility assays, majority of the cells present in those tubes were daughter generations of the HF-exposed original inoculum but themselves were never directly exposed to HF.

Susceptibility of *S. marcescens* to all four test antibiotics was decreased owing to HF treatment. This suggests that if a patient is simultaneously taking allopathic and traditional medicinal agents, then wrong combinations of antibiotics and plant extracts can be harmful. In our study, we did not investigate the possibility of synergistic action of HF and antibiotics, as the test bacteria were not challenged with the HF-antibiotic combinations. However, it is not uncommon in real-world situations for patients to take conventional antibiotics as prescribed by an allopathic doctor, with simultaneous self-motivated intake of some herbal products (not usually informed to the doctor). Here, there is a probability of the pharmacological effects of the allopathic drug and the herbal product being antagonistic, which may result in either delayed patient recovery or even undesired side effects (https://www.pharmatutor.org/articles/herbs-interaction-allopathic-drugs-review).

HF was found to enhance catalase activity by 8–20% in all the gram-negative bacteria. It may be speculated that HF exposure might induce oxidative stress in these bacteria, and to counteract this elevated oxidative stress, the bacteria are forced to overwork their oxidative stress response machinery, of which catalase is an important component. Oxidative stress stems from the reactive oxygen species (ROS), and these ROS have been indicated as modulators of bacterial virulence [48]. Induction of redox-associated physiological alterations has been reported to be a part of the overall antibiotic effect exerted by conventional antibiotics [49]. Among the antibiotics employed by us as controls in this study, sub-MIC concentrations of tetracycline also induced catalase activity of *P. aeruginosa* and *S. marcescens* by 23.68% and 41.66%, respectively (data not shown). Generation of ROS has been suggested as a unifying mechanism of diverse antibiotics, and increasing importance is being attached to redox mechanisms in the context of antibiotic activation and resistance [50]. Infectious pathogens such as *Escherichia coli*, *Staphylococcus aureus*, and *Mycobacterium tuberculosis* are considered to be sensitive to changes in the intracellular oxidative environment. Thus, formulations that disturb the cellular oxidative environment can serve as novel therapeutics [51].

Haemolytic activity of all three bacteria was compromised by ∼18–68% upon HF exposure. Hemolysis is considered an important virulence trait of many pathogens, as it allows the pathogenic bacteria access to the otherwise bound iron [52]. Iron requirement of pathogens inside the host far exceeds the amount of free iron present in human serum, and hence, therapeutic agents capable of compromising the haemolytic potential of bacteria can make their survival and replication inside the host difficult. HF-exposed *P. aeruginosa* was observed to produce lesser pyoverdine, which is an iron scavenger. This observation is important in light of the fact that *P. aeruginosa* wound infection is reported to involve activation of its iron acquisition system in response to fascial contact, and appropriate iron availability is also necessary for the bacteria to maintain a high population density in the wound [10].

HF at 0.5% v/v was found to increase the susceptibility of *P. aeruginosa* to human serum by 25%. This property of antimicrobial formulations like HF can aid the host immune system in rapid clearance of bacteria from the body, as lysis by serum will leave lesser bacteria to be dealt with by the body's defense mechanisms. Influences of the serum on expression of *P. aeruginosa* QS- and virulence-associated genes and QS-controlled virulence genes were investigated in [53]. They showed that, at early stages of growth, serum can repress the expression of many *P. aeruginosa* genes, and it can enhance them in the late phase. HF's ability to enhance *P. aeruginosa* susceptibility to human serum becomes even more important in light of the fact that serum-resistant phenotypes of *P. aeruginosa* are more frequently isolated from wounds and blood than infections at other sites [54]. Wound infections are believed to occur when microbial burden exceeds the innate clearance capacity of the host immune system [10]. Haemolysin and serum-resistance profile of clinical isolates are considered important aspects to be investigated [55].

HF at 0.5% v/v inhibited biofilm formation of *P. aeruginosa* by nearly 40%. This corroborates well with 9.32% reduction in CSH of this bacterium upon HF exposure. Surface hydrophobicity is an important determinant of bacteria's ability to form biofilm [32], and hydrophobic cells are believed to form stronger biofilms on medical implants constructed from hydrophobic materials [56]. Tribedi and Sil [57] indicated a direct correlation between CSH of *Pseudomonas* sp. and its ability to degrade nonpolar polymers. Gram-negative bacteria increase their CSH by releasing membrane vesicles, when subjected to stressful conditions [58]. CSH modification is considered to be a part of bacterial adaptive modifications in face of environmental changes. Reduced hydrophobicity may interfere with clumping of the bacterial cells by promoting the intercellular repulsion [59] and thus reduce biofilm formation.

**Figure 6:**
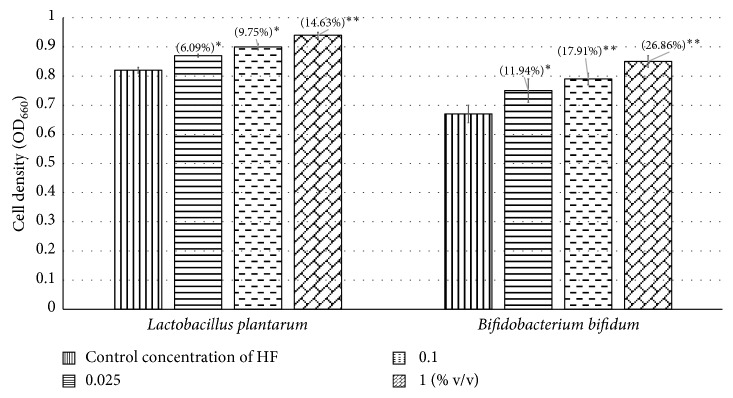
Growth-promoting effect of HF on probiotic strains. ^*∗*^ *p* < 0.05; ^*∗∗*^ *p* < 0.01. Bacterial growth was measured as OD_660_.

We have demonstrated Herboheal to be effective against three different gram-negative bacteria. In general, it is difficult to find “hits” against gram-negative bacteria owing to presence of the outer membrane in their cell surface, which poses an additional entry barrier for majority of antimicrobials [60]. Gram-negative infections are believed to predominate in burn surgery, and *P. aeruginosa* is among one of the most common such burn wound pathogens [61]. In recent years, use of probiotic strains has also been indicated as a promising strategy (referred to as bacteriotherapy, or replacement therapy, or bacterial interference) for management of chronic wounds, wherein probiotic bacteria like lactobacilli are directly applied onto infected wound [62]; we investigated the effect of HF on two probiotic strains, namely, *L. plantarum* and *B. bifidum*. HF (at 0.025–1% v/v) could enhance growth of these bacteria by 6–27% (Figure 6). *L. plantarum* has earlier been reported for its antipathogenic properties against *P. aeruginosa* by Ramos et al. [63], pointing to the potential use of its supernatants for treating infected chronic wounds. Similarly, Gan et al. [64] reported successful use of *L. fermentum* RC-14 and its secreted products in inhibiting surgical implant infections involving *S. aureus.* With lactobacilli being present in abundance in the vaginal tract, applying wound-care formulations with prebiotic (i.e., promoting growth of probiotic strains) properties on vaginal rashes/cuts and perineal wounds may help achieve faster healing. Such formulations may be effective in dealing with vaginal dysbiosis associated with bacterial vaginitis, at which current treatment options have limited success [65].

## 5. Conclusions

**Figure 7:**
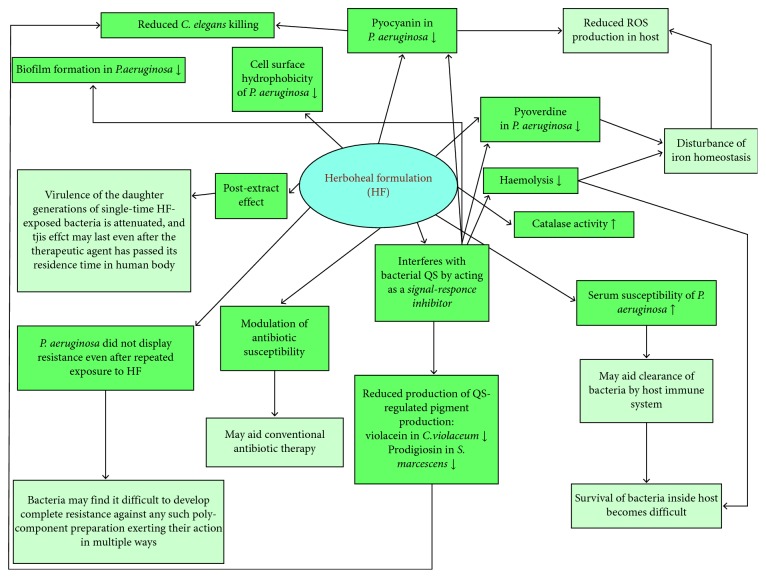
A summarized picture of the antipathogenic effect of Herboheal formulation against three different gram-negative bacterial pathogens.

A summarized picture of the antipathogenic effect of the polyherbal Herboheal formulation investigated in this study against three different gram-negative bacterial pathogens is presented in Figure 7. The test formulation used in this study is a polyherbal formulation, and traditional medicine whether Indian, Arabian, or Chinese has always heavily relied on polyherbal and herbomineral formulations, which do not solely rely on activity of any single constituent. Their inherent polycomponent nature makes them less prone to development of resistance by the susceptible microorganisms, as for any organism developing simultaneous resistance against multiple components will always be more difficult than that against a single-molecule-based agent. We recently have reported antipathogenic potential of another polyherbal formulation described as *Panchvalkal* in *Ayurved* [16]. This concept of polyherbalism/multicomponent formulations is gaining wider acceptance today than ever before [66], and accordingly, there is an increasing interest among scientific community to validate traditional therapeutic practices. Generation of scientific evidence to verify the claims made regarding traditional medicine will make it more acceptable in the modern world. The current study is an example of such validation exercises, wherein we have shown the efficacy of Herboheal against three such bacteria which are known to be involved in wound infections. Further investigation regarding how the transcriptional and translational profiles of these bacteria change under the influence of Herboheal can explain the molecular mechanisms of its antipathogenic efficacy.

## Data Availability

All data pertaining to this study have been included within the manuscript.

## Supplementary Materials

Supplementary Materials Appendix A: information on development and composition of the test formulation “Herboheal.” Table S1: a comparison of efficacy of HF (0.1% v/v) when it is used against *P. aeruginosa* before infecting *C. elegans* versus when it is used on *C. elegans* already infected by *P. aeruginosa* having no previous exposure to the HF.

## Abbreviations

QS: Quorum sensing
HF: Herboheal formulation
PEE: Post-extract effect
CSH: Cell surface hydrophobicity
AS: Antibiotic susceptibility.
