# Supplementary material for: Antipathogenic Potential of a Polyherbal Wound-Care Formulation (Herboheal) against Certain Wound-Infective Gram-Negative Bacteria
Source: Adv Pharmacol Sci. 2019 Jan 27;2019:1739868. doi: 10.1155/2019/1739868 (PMC6369513; doi:10.1155/2019/1739868)
Supplement: Supplementary Materials — Appendix A: information on development and composition of the test formulation “Herboheal.” Table S1: a comparison of efficacy of HF (0.1% v/v) when it is used against P. aeruginosa before infecting C. elegans versus when it is used on C. elegans already infected by P. aeruginosa having no previous exposure to the HF. [file 1739868.f1.pdf]

Supplemental information

Appendix A: Supplementary information on development and composition of the test formulation 'Herboheal'

Herboheal formulation (License No. GA/1616) was procured from the SRISTI organization, Ahmedabad. Brief notes on this formulation can be seen at: http://www.sristiinnovation.com/human-products-best-for-your-skin.html#herboheal-herbal-wound-healing-ointment. Further details on this formulation including its composition has been provided in the supplementary file (Appendix A). The practices related to the formulation of Herboheal for wound healing was developed at SRISTI's Natural Products Laboratory [Sadbhav SRISTI Sanshodhan Natural Product Laboratory (SSSNPL)] by pooling various grassroot practices. SSSNPL is involved in bridging the gap between informal and formal knowledge to develop the products based on grassroots practices. These practices were documented by SRISTI and being managed as a database. The open source database of such knowledge can be seen at: (http://www.sristi.org/wsa/). Name of knowledge holders as mentioned on the product are: Devkaranbhai L. Rabari, Naranbhai D. Rabari, Jagataben Damor, Lilaji M. Parmar, Becharbhai S. Devgania, and Haribhai M. Narolla. Composition of the test formulation (Herboheal cream) obtained from SRISTI is presented below:

Composition of the test formulation

Scientific name of the plant	Common name (English/ Hindi)	Plant part used in formulation	Proportion in overall composition
Azadirachta indica A.Juss.	Neem	Leaves	14%
Acacia nilotica	Babul	Bark	14%
Ocimum sanctum L.	Holy Basil/ Tulsi	Leaves	28%
Annona squamosa L.	Custard apple/ Sitaphal	Leaves	28%
Curcuma longa L.	Turmeric/ Haldi	Rhizome	7%
Ricinus communis L.	Castor oil plant	Seed oil	Quantum satis (q.s.)
Bee wax		-	q.s.

Plant names listed above have been checked with http://www.theplantlist.org on May 20, 2018.

Table S1: A comparison of efficacy of HF (0.1%v/v) when it is used against *P. aeruginosa* before infecting *C. elegnas*, versus, when it is used on *C. elegans* already infected by *P. aeruginosa* having no previous exposure to the HF

Mode of HF application	% Survival of worm population at the end of 5-day experiment (Mean ± SD)	Net survival benefit at the end of 5-day experiment (after nullifying natural death of few worms in 'control' wells) (%) (Mean ± SD)
**Pre-treatment of bacteria with HF before allowing them to infect *C. elegans***	**67.5 ± 2.50**	**[a]55*** ± 3.49**
**HF used as a therapy for already infected *C. elegans* (6h post-infection)**	**63 ± 1.73**	**[b]50.5*** ± 3.12**
**HF used as a therapy for already infected *C. elegans* (24h post-infection)**	**31.33 ± 3.51**	**[c]18.83** ± 5.00**

HF: Herboheal formulation;

p value pertaining to difference in the survival values between 'a' and 'b': 0.06; *p* value pertaining to difference in the survival values between 'b' and 'c': 0.0001; *p* value pertaining to difference in the survival values between 'a' and 'c': 0.001. Proportion of living worms in the 'control' wells at the end of fifth day was 12.5 ± 1.5%.
